# Comparative proteomic analysis of multi-ovary wheat under heterogeneous cytoplasm suppression

**DOI:** 10.1186/s12870-019-1778-y

**Published:** 2019-05-02

**Authors:** Jialin Guo, Gaisheng Zhang, Yulong Song, Zheng Li, Shoucai Ma, Na Niu, Junwei Wang

**Affiliations:** College of Agronomy, National Yangling Agriculture Biotechnology & Breeding Center, Yangling Branch of State Wheat Improvement Centre, Wheat Breeding Engineering Research Center, Ministry of Education, Key Laboratory of Crop Heterosis of Shaanxi Province, Northwest A & F University, Yangling, 712100 Shaanxi China

**Keywords:** Floral organ development, Multi-ovary, Nuclear-cytoplasm interaction, Proteomics, *Triticum aestivum* L

## Abstract

**Background:**

DUOII is a multi-ovary wheat (*Triticum aestivum* L.) line with two or three pistils and three stamens in each floret. The multi-ovary trait of DUOII is controlled by a dominant gene, whose expression can be suppressed by the heterogeneous cytoplasm of TeZhiI (TZI), a line with the nucleus of common wheat and the cytoplasm of *Aegilops*. Crosses between female DUOII plants and male TZI plants resulted in multi-ovary F_1_s; whereas, the reciprocal crosses resulted in mono-ovary F_1_s. Although the multi-ovary trait is inherited as single trait controlled by a dominant allele in lines with a *Triticum* cytoplasm, the mechanism by which the special heterogeneous cytoplasm suppresses the expression of multi-ovary is not well understood.

**Results:**

Observing the developmental process, we found that the critical stage of additional pistil primordium development was when the young spikes were 2–6 mm long. Then, we compared the quantitative proteomic profiles of 2–6 mm long young spikes obtained from the reciprocal crosses between DUOII and TZI. A total of 90 differentially expressed proteins were identified and analyzed based on their biological functions. These proteins had obvious functional pathways mainly implicated in chloroplast metabolism, nuclear and cell division, plant respiration, protein metabolism, and flower development. Importantly, we identified two key proteins, Flowering Locus K Homology Domain and PEPPER, which are known to play an essential role in the specification of pistil organ identity. By drawing relationships between the 90 differentially expressed proteins, we found that these proteins revealed a complex network which is associated with multi-ovary gene expression under heterogeneous cytoplasmic suppression.

**Conclusions:**

Our proteomic analysis has identified certain differentially expressed proteins in 2–6 mm long young spikes, which was the critical stage of additional primordium development. This paper provided a universal proteomic profiling involved in the cytoplasmic suppression of wheat floral meristems; and our findings have laid a solid foundation for further mechanistic studies on the underlying mechanisms that control the heterogeneous cytoplasm-induced suppression of the nuclear multi-ovary gene in wheat.

**Electronic supplementary material:**

The online version of this article (10.1186/s12870-019-1778-y) contains supplementary material, which is available to authorized users.

## Background

Wheat (*Triticum aestivum* L.), the largest grain crop in the world, accounts for about 26% of global grain production and 44% of cereals used for food. As an important staple food, wheat provides around 20% of the protein, 18% of the calories, and 3% of the fat consumed by the human population [1]. With an increasing world population and decreasing cultivated land, there is a need to increase wheat yield per unit area to ensure world food security. Generally, there is only one seed in each floret of wheat. However, Chen et al. [2] discovered and cultivated “trigrain wheat”, which has two or three pistils and three stamens, and results in two to three seeds per floret. This multi-ovary trait of wheat is genetically stable and has the obvious advantage of increased number of grains per spike; therefore, multi-ovary wheat offers an excellent opportunity to study floral developmental mechanisms and to increase wheat yield.

Since the wheat multi-ovary trait was reported in 1983, studies have mainly focused on the developmental process of floral organs [3], biochemical basis of seed germination [4], discovery of molecular markers [5], gene localization and genetic analysis [6], and mechanisms of multi-ovary development [7, 8]. In a previous study, we found that the multi-ovary trait of DUOII is controlled by a dominant gene, and that the F_1_s derived from the reciprocal crosses between DUOII and TeZhiI (TZI, a alloplasmic line with the nucleus of common wheat and the cytoplasm of *Aegilops*) are phenotypically different. Specifically, the cross between a female DUOII and a male TZI produces a multi-ovary F_1_; in contrast, the cross between a female TZI and a male DUOII produces a mono-ovary F_1_. In addition, in the F_2_ generation, the multi-ovary trait segregates 3:1 in both crosses, however it segregates as a dominant allele in the DUOII (♀) × TZI (♂) cross, whereas it segregates as a recessive allele in the TZI (♀) × DUOII (♂) cross. As the nuclear DNA involved is the same in both crosses, the multi-ovary trait may be determined by a nuclear-cytoplasmic interaction, and the expression of the multi-ovary gene may be suppressed by the heterogeneous cytoplasm of TZI [9]. However, information on the underlying mechanism that controls the heterogeneous cytoplasmic suppression of the multi-ovary gene in common wheat is limited.

The normal development of floral organs depends on the intricate and precise regulation of gene expression. Proteins, the gene products, are the main and direct executors of cellular functions and reflect the complex molecular and physiological processes that operate in plants [10, 11]. Although each cell of an organism has the same genome, the protein contents of individual cells and tissues show considerable differences [12–14]. Proteomics, the large-scale analysis of proteins, has contributed greatly to our understanding of gene function in the post-genomic era [15]. In addition, the completion of wheat genome sequencing has greatly promoted the application of proteomics in wheat research [16–19]. Therefore, proteomics has become a pivotal tool to unravel the molecular mechanisms of complex biological processes at the protein level in wheat and has been successfully applied in various processes, such as grain development [20], lateral meristem development of branched spikes [21], male sterility [22], and responses to different biotic and abiotic stresses [23–25].

In this study, we applied a two-dimensional electrophoresis (2-DE) based mass spectrometry (MS) approach to investigate comparative alterations of protein profiles in the F_1_s derived from reciprocal crosses between DUOII and TZI plants, and a considerable number of proteins were identified to show differences in their relative abundances in these two crosses. The proteomic results provided new insight into the underlying molecular mechanisms that control the heterogeneous cytoplasmic suppression of the multi-ovary gene in wheat. The findings will provide a foundation for understanding the development of the multi-ovary trait in wheat, and can be implemented in future breeding activities focused on the development of high yield wheat cultivars.

## Methods

### Plant materials

In this study, we used two inbred wheat lines: DUOII, which is a common multi-ovary line, and TZI, which is an alloplasmic line with the nucleus of wheat variety ‘Chris’ and the cytoplasm of an *Aegilops* species. Plant seeds of these two lines were obtained from Key Laboratory of Crop Heterosis of Shaanxi Province, Northwest A & F University, Shaanxi, China. Prior to initiating the study, these two lines had been selfed for more than twenty years and no segregation occurred in any aspect, which ensured their genetic backgrounds pure enough. In October 2014, DUOII and TZI were sown at the experimental field of Northwest A & F University, Yangling, China (34°91’N, 106°86’E). In May 2015, reciprocal crosses between DUOII and TZI were performed, and the F_1_ seeds were sown in October 2015. In March 2016, 2–6 mm young spikes were hand dissected from approximately 90 plants in each F_1_ population (DUOII (♀) × TZI (♂) and TZI (♀) × DUOII (♂)), immediately frozen in liquid nitrogen, and stored at − 80 °C until protein extraction.

### Morphological analysis and cytological examination

The agronomic traits of F_1_ individuals were measured on five plants randomly selected from the reciprocal crosses, and all measurements were done according to [26]. Plant height was measured from the ground to the top of the spike, and the spike number per plant, spikelet number on the main stem spike, seed number on the main stem spike, seed number per spikelet of the main stem, and thousand seed weight were investigated. The mean values were used to characterize the corresponding traits. Photographs of F_1_ spikes were obtained using a Nikon D600 digital camera (Nikon, Tokyo, Japan), and photographs of pistils were obtained using a Nikon E995 digital camera (Nikon) mounted on a Motic K400 dissecting microscope (Preiser Scientific, Louisville, KY, USA). For cytological examination, young spikes were processed as described by [27] and observed with a JSM-6360LV scanning electron microscope (JEOL, Tokyo, Japan).

### Young spike protein extraction and quantification

Since the different length of young spikes are at different developmental stages, in order to confirm the samples was same between DUOII × TZI and TZI × DUOII, approximately 1 g of frozen young spikes collected equally from 2 to 3 mm, 3–4 mm, 4–5 mm and 5–6 mm young spikes were mixed as one biological replicate. Proteins were extracted from three biological replicates using the trichloroacetic acid (TCA)-acetone method as described by [28] with a few modifications. In brief, frozen young spikes from three biological replicates were separately ground into a fine powder in liquid nitrogen using a sterilized and chilled pestle and mortar, homogenized with pre-cooled 10% (w/v) TCA/acetone containing 0.07% (v/v) 2-mercaptoethanol (2-ME) and 1 mM phenylmethanesulfonyl fluoride (PMSF), and subsequently precipitated overnight at − 20 °C. Precipitated proteins were centrifuged at 20,000×g for 30 min at 4 °C with the supernatant fluid discarded. Then, the pellets were suspended in pre-cooled acetone containing 0.07% (v/v) 2-ME and 1 mM PMSF, placed at − 20 °C for 1 h, and centrifuged at 20,000×g for 30 min. After repeating this procedure four times, the precipitates were vacuum-dried. The dried pellets were subsequently resuspended in lysis buffer containing 7 M urea, 2 M thiourea, 4% (w/v) 3-[(3-cholamidopropyl) dimethylammonio] propanesulfonate (CHAPS), 65 mM DL-dithiothreitol (DTT), 0.5% (v/v) Bio-Lyte (pH 4–7), and 0.0001% (w/v) bromphenol blue. After vortexing for 2 min at room temperature, the suspension was incubated for 30 min at 29 °C, and then frozen in liquid nitrogen. This procedure was repeated three times, and the suspension was centrifuged at 18,000×g for 30 min at 23 °C to collect the supernatant fluid. The protein concentration of the final supernatant was determined according to the method of Bradford [29] with bovine serum albumin as a standard. The proteins were stored at − 80 °C for 2-DE analysis.

### 2-DE and image analysis

2-DE was performed according to previously established procedures [30]. For isoelectric focusing (IEF), about 900 μg of protein sample was loaded on a ReadyStrip™ IPG Strip (17 cm, pH 4–7, Bio-Rad, USA) and rehydrated passively at 50 V for 14 h at 20 °C using a PROTEAN IEF Cell (Bio-Rad, USA). The subsequent focusing procedure was performed over six steps: 1) increasing linearly from 50 to 250 V for 1 h; 2) increasing linearly from 250 to 500 V for 1 h; 3) increasing fast to 1000 V and maintained for 1 h; 4) increasing linearly from 1000 to 8000 V for 4 h; 5) maintained at 8000 V for a total of 80,000 Vh; 6) reducing fast to a constant 500 V until the next step. After IEF, the focused strips were incubated for 15 min with gentle shaking in “equilibration buffer I” consisting of 6 M urea, 2% (w/v) sodium dodecyl sulfate (SDS), 0.375 M Tris-HCl (pH 8.8), 20% (v/v) glycerol, 2% (w/v) DTT, and then for another 15 min in “equilibration buffer II,” which contained 2.5% (w/v) iodoacetamide instead of the 2% (w/v) DTT in “equilibration buffer I.” For second dimension electrophoresis, the strips were transferred to the top of 12% vertical sodium dodecyl sulfate-polyacrylamide gel electrophoresis (SDS-PAGE) gels. Electrophoresis was run at 15 °C and at 10 mA per gel for 1 h, followed by 20 mA per gel until the bromophenol blue dye front reached the base of gel.

After SDS-PAGE, the gels were stained with Coomassie brilliant blue (CBB) G250 solution, and visualized at a resolution of 600 dpi using a Powerlook 2100XL imaging densitometer (UMAX, Taiwan, China). Image analysis was performed using the analytical software PDQuest 8.0.1 (Bio-Rad, USA) according to the manufacturer’s instructions. Protein spots on the gels were detected and quantified, and the background was subtracted. Only protein spots expressed by a ≥ 1.5-fold change with a *P* < 0.05 were considered as differentially expressed proteins (DEPs).

### In-gel digestion and LC-HESI-MS/MS analysis

Protein spots were manually excised and destained repeatedly with fresh solution consisting of 50% acetonitrile (ACN) in 25 mM ammonium bicarbonate (ABC) buffer until the blue color disappeared. Next, the protein spots were washed with Milli-Q water and subsequently dehydrated with 500 μL 100% ACN. Disulfide bonds were reduced by incubating the protein spots for 1 h at 56 °C with 200 μL of 10 mM DTT in 25 mM ABC buffer. The alkylation of cysteines was carried out with 200 μL of 55 mM iodoacetamide in 25 mM ABC buffer by incubating for 45 min at room temperature in darkness. Gel pieces were washed twice using 25 mM ABC and dehydrated with pure ACN. Then, the dried gel pieces were incubated with 10 ng/μL trypsin (sequencing-grade reagent, Promega, USA) solution in 25 mM ABC in an ice bath for 30 min, and then transferred into a 37 °C incubator for digestion overnight. Following enzymatic digestion, the supernatant was collected and the peptides of gels were extracted once by 200 μL 0.1% formic acid (FA) in 50% ACN and twice by 200 μL 0.1% FA in pure ACN. All the supernatant was combined and dried completely using a vacuum centrifuge.

Each dried peptide sample was resuspended in solvent A (2% ACN, 0.1% FA) and centrifuged at 20,000×g for 10 min. The supernatant was collected for LC-HESI-MS/MS (Liquid chromatography with heated electrospray ionization tandem mass spectrometry) analysis. First, the peptide sample was loaded on an LC-20 AD nanoHPLC (Shimadzu, Japan) equipped with a Cap-trap column and eluted with solvent A at 15 μL/min for 4 min. Then, the eluate was separated in a 10-cm C18 reverse-phase analytical capillary column (inner diameter 75 μm) made in house. The chromatographic gradient was run at 400 nL/min with solvent B (98% ACN, 0.1% FA) starting from 2 to 35% for 44 min, followed by from 35 to 80% for 5 min, maintained at 80% for 4 min, and finally 5% for 1 min. The peptide samples were subject to heated electrospray ionization tandem mass spectrometry in an LTQ Orbitrap Velos (Thermo Fisher, USA) coupled to the HPLC (High performance liquid chromatography). The mass spectrometer was operated in data-dependent mode, automatically switching between MS and MS/MS. Following a survey MS scan (350–1500 *m/z*) with a resolution of 30,000 in the Orbitrap analyzer, the top six most intense ions were selected for MS/MS analysis in high-energy collision-induced dissociation (HCD) mode at a normalized collision energy of 27 V and a resolution of 7500.

### Protein identification

Raw MS/MS spectra data were converted into MGF files using Proteome Discoverer software (version 1.2, Thermo Fisher, USA). Peptide identification of MGF files were performed using the MOSCOT search engine (http://www.matrixscience.com) against the NCBInr and Swiss-Prot databases with a taxonomy parameter set to viridiplantae (6,686,534 sequences and 39,582 sequences). The database search parameters were as follows: trypsin digest with one missed cleavage, peptide mass tolerance of 20 ppm, MS/MS tolerance of 0.1 Da, peptide charge of 1+, carbamidomethyl (C) as fixed modifications, and oxidation (M), Gln- > pyro-Glu (N-term Q), deamidated (NQ) as variable modifications. The MASCOT score, the number of peptides matched, the number of sequences matched, isoelectric point (pI), and molecular weight (Mr) were used to evaluate the database searching results.

### Bioinformatics analysis

The differentially expressed proteins were subjected to Gene Ontology (GO) and Kyoto Encyclopedia of Genes and Genomes (KEGG) analysis using the website-based tool agriGO (http://bioinfo.cau.edu.cn/agriGO/) and KOBAS (version 3.0; http://kobas.cbi.pku.edu.cn). All identified proteins were blasted against the TAIR (the *Arabidopsis* Information Resource) protein databases and used for constructing a protein-protein interaction (PPI) network with the online analysis tool STRING 10.0 (http://string-db.org). In addition, IBM SPSS Statistics 20.0 (IBM Crop., Armonk, NY, USA) and Microsoft Excel 2016 (Microsoft Crop., Redmond, WA, USA) were used to process all other data. The differential expression of protein spots was determined by Student’s *t*-test at a significance level of 0.05. The data of phenotypic traits were analyzed through variance analysis method and multi-ovary comparisons among the mean values were confirmed using Least-Significant Difference (LSD) test at a significance level of 0.05.

## Results

### Phenotypic differences between DUOII × TZI and TZI × DUOII plants

**Fig. 1:**
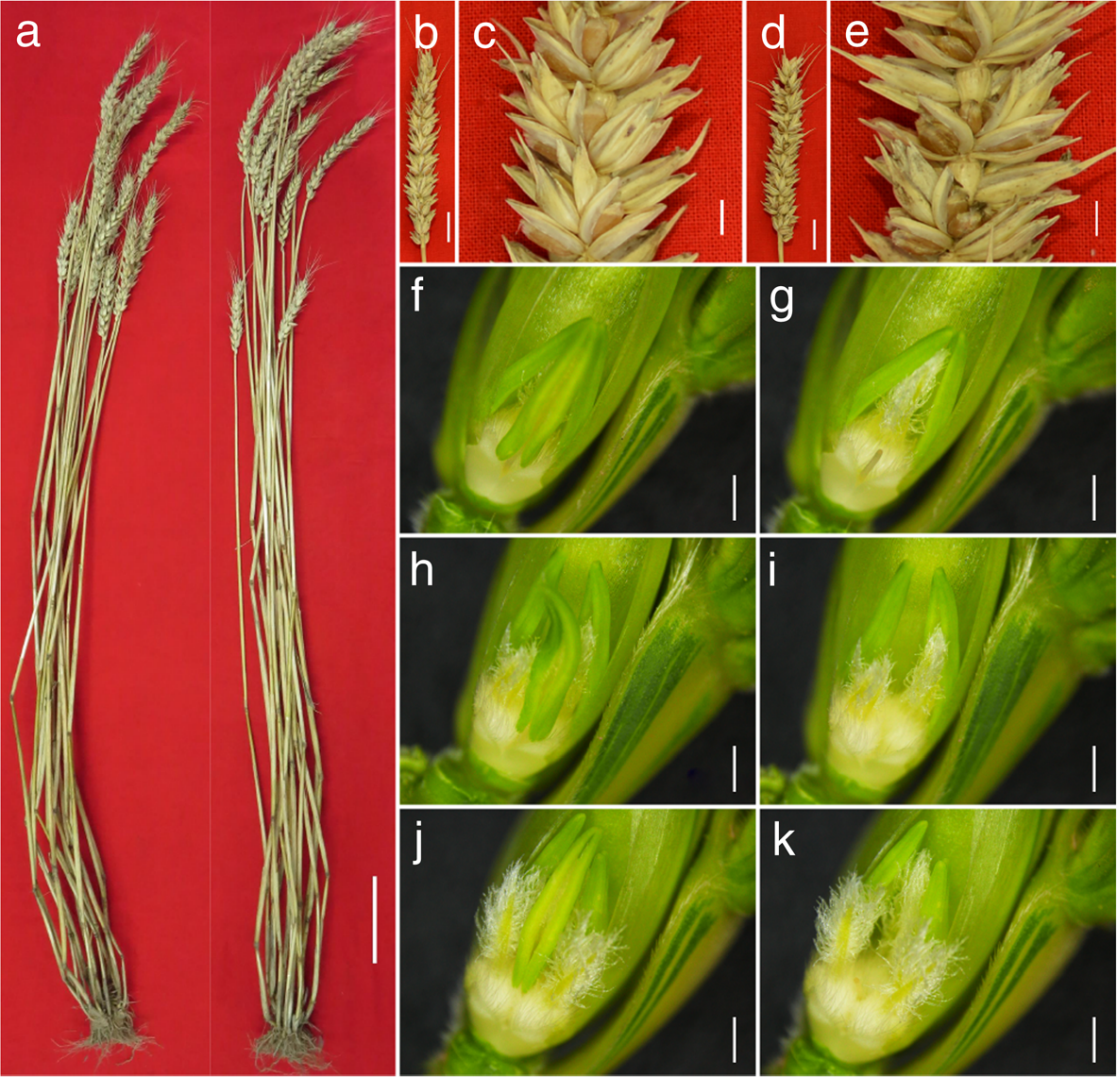
Phenotypic characterization of TZI × DUOII and DUOII × TZI crosses. **a** The morphology of TZI × DUOII (left) and DUOII × TZI (right) plant. **b** The spike of TZI × DUOII plant. **c** Enlarged view of TZI × DUOII spike. **d** The spike of DUOII × TZI plant. **e** Enlarged view of DUOII × TZI spike. **f, g** One ovary in one floret. **h, i** Two ovaries in one floret. **j, k** Three ovaries in one floret. Bars = 10 cm in (**a**), 2 cm in (**b, d**), 5 mm in (**c, e**), 1 mm in (**f-k**)

For the reciprocal crosses between multi-ovary wheat DUOII and alloplasmic cytoplasm wheat TZI, the phenotypic characteristics of F_1_ plants were similar; however, the multi-ovary trait was different (Fig. 1). All 50 DUOII × TZI plants expressed the multi-ovary trait; whereas, all 50 TZI × DUOII plants expressed the mono-ovary trait. In addition, there were no significant differences in plant height, spike length, spike number, and spikelet number on the main stem spike between the reciprocal crosses. Seed number per spikelet of the main stem of the DUOII × TZI plants was significantly higher than that of the TZI × DUOII plants, as was the seed number on the main stem spike; while the thousand seed weight of DUOII × TZI plants was significantly lower than that of the TZI × DUOII plants (Table 1). All of these significant differences resulted from the multi-ovary trait, so the results showed that the heterogeneous cytoplasm only influenced the expression of the multi-ovary-related traits, but not influenced the other morphological traits.

**Table 1:** Characteristics of F1 plants and their parents

Traits	DUOII	TZI	DUOII × TZI	TZI × DUOII
Plant height (cm)	94.2 ± 2.8^c^	136.0 ± 3.2^a^	127.6 ± 3.6^b^	125.6 ± 7.0^b^
Spike length (cm)	8.9 ± 0.5^b^	11.4 ± 0.6^a^	12.4 ± 0.5^a^	11.3 ± 0.7^a^
Spike number	10.0 ± 2.0^c^	14.4 ± 2.1^bc^	20.8 ± 4.7^a^	16.6 ± 2.5^ab^
Spikelet number on the main stem spike	21.4 ± 1.1^a^	19.4 ± 1.1^b^	19.6 ± 0.9^ab^	19.2 ± 1.1^b^
Seed number on the main stem spike	120.2 ± 7.3^a^	41.0 ± 6.0^d^	104.8 ± 7.1^b^	60.4 ± 4.7^c^
Seed number per spikelet of the main stem	5.6 ± 0.2^a^	2.1 ± 0.2^b^	5.4 ± 0.3^a^	3.1 ± 0.2^b^
Thousand seed weight (g)	22.8 ± 0.5^c^	44.0 ± 0.2^a^	32.9 ± 1.0^b^	43.3 ± 0.5^a^

Data were presented as mean ± SD and the same letter(s) in a row were not significantly different at *P* = 0.05

### Morphological characterization of additional pistil development

**Fig. 2:**
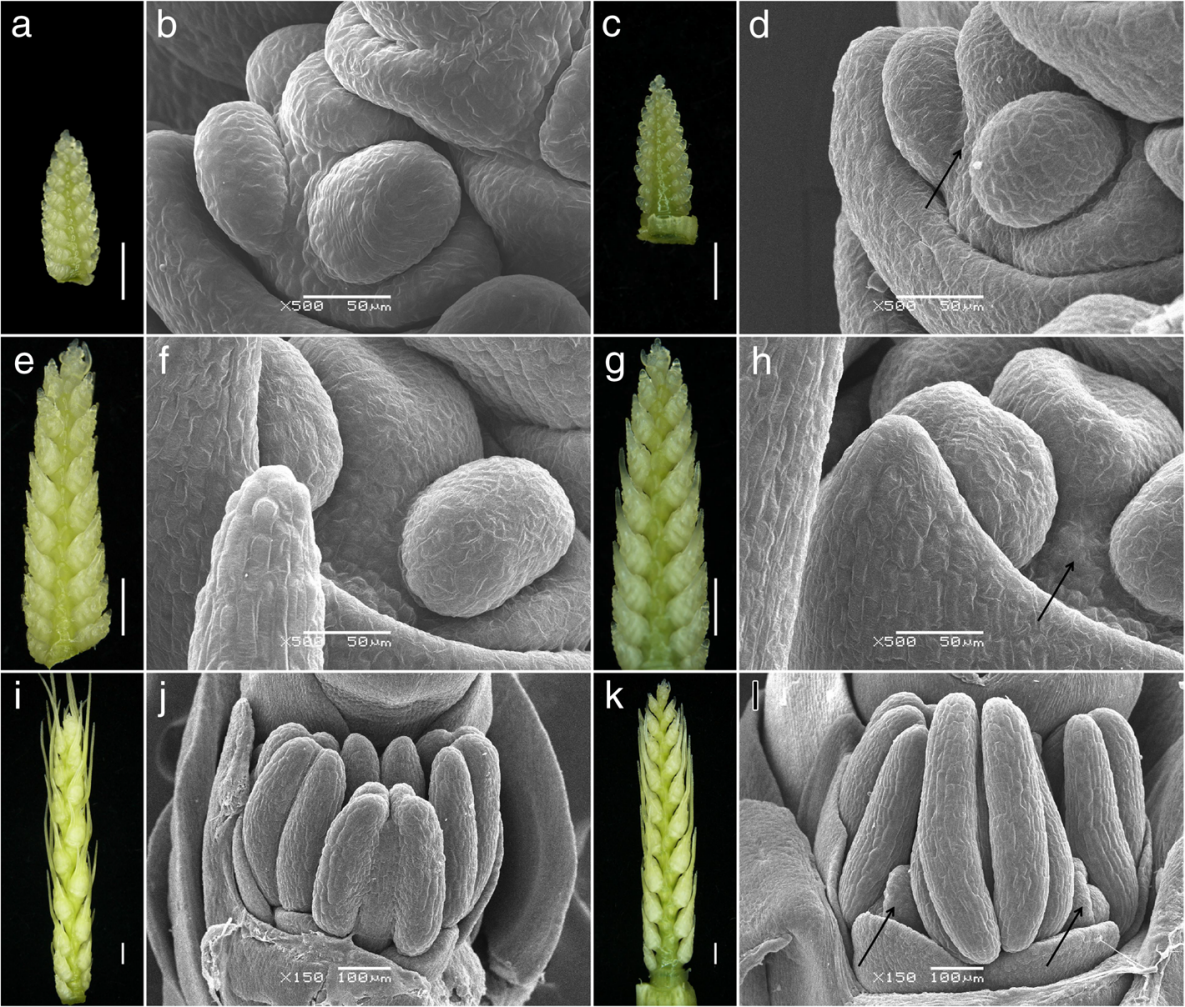
Comparative morphological characteristics of multi-ovary and mono-ovary young spikes. **a-d** 2–3 mm young spikes. **e-h** 5–6 mm young spikes. **i-l** about 15 mm young spikes. **a, b, e, f, i, j** mono-ovary wheat. **c, d, g, h, k, l** multi-ovary wheat. Arrow indicates the position of additional pistil. Bars = 1 mm in (**a, c, e, g, i, k**), 50 μm in (**b, d, f, h**), 100 μm in (**j, l**)

In the middle floret of the 2–3 mm long young spikes of DUOII, a small and not very obvious protuberance was generated at the base of the main pistil between the frontal stamen and lateral stamen (Fig. 2c-d), which was considered as the first sign of the additional pistil. During spike development, the protuberance increased in size and became obvious in the middle floret of the 5–6 mm long young spikes (Fig. 2g-h). In order to confirm whether this protuberance would continually grow into a pistil, we observed the development state of this protuberance when the spike grew to about 15 mm. This protuberance became bigger, and its position was the same as the final additional pistil (Fig. 2k-l). Through continuous observation of the development of this protuberance, it did develop to an additional pistil (Fig. 1h-k). As a control, no protuberance was observed in the same position of common wheat (Fig. 2a-b, e-f, i-j). So, this protuberance was the original form of the additional pistil, and the first observed stage of the additional pistil was identified when the young spikes were 2–6 mm long, which corresponded to the standard developmental stage 4.25–4.5 according to Waddington scale [31].

### Proteome profiles during the development of an additional pistil

To investigate the proteomic differences in the young spikes of TZI × DUOII and DUOII × TZI plants, proteins of 2–6 mm spikes from these reciprocal crosses were extracted and independently separated by 2-DE analysis. According to the 2-DE protein maps (Additional file 1: Figure S1), the TZI × DUOII and DUOII × TZI plants had similar proteomic profiles, and there were about 800 gel spots detected over the gels. Due to the sensitivity and reproducibility of 2-DE technology, we used a ≥ 1.5-fold difference in expression with *P* < 0.05 as a selection criterion to identify the DEPs. Compared to the DUOII × TZI plants, a total of 90 gel spots were identified as DEPs in the TZI × DUOII plants, of which 72 proteins were downregulated and 18 were upregulated in the TZI × DUOII plants relative to DUOII × TZI (Additional file 2: Figure S2; Additional file 3: Table S1). Among the 90 DEPs, 85 proteins were co-expressed in both of the two crosses, and only one protein (spot 37) and four proteins (spot 56, spot 64, spot 81, spot 87) were specifically expressed in the DUOII × TZI and TZI × DUOII plants, respectively. These DEPs may possibly be related to the mechanism of heterogeneous cytoplasmic suppression of the multi-ovary gene.

### Identification of DEPs

To identify these DEPs, all of which were analyzed by LC-HESI-MS/MS analysis, and were successfully identified by searching against the NCBInr and Swiss-Prot databases with MASCOT tool. Among the 90 spots, 87 have been functionally annotated in the current database, whereas the remaining three spots were either unnamed proteins (spot 29, spot 87) or predicted proteins (spot 19) (Additional file 3: Table S1). To annotate these spots, we used the sequences of these spots as a query to search for homologs by BLASTP (NCBI), and the corresponding homologs with the highest similarities are listed in Additional file 4: Table S2. These three proteins shared at least 80% sequence similarity, suggesting that they may have similar function with their homologs. In addition, of the 90 DEPs, some candidate proteins from different positions of the same gel and with the different Mr. and pI were expected to have the same name, e.g., ATP synthase subunit alpha (spot 66, spot 76), flowering locus K homology domain (spot 25, spot 26, spot 27), and transketolase (spot 50, spot 55). These spots may be different isoforms of candidate proteins due to nucleotide polymorphism, alternative splicing, or post-translational modifications, which might thus have different biological functions. In summary, 90 DEPs represented 82 unique proteins.

### GO analysis and KEGG enrichment of DEPs

**Fig. 3:**
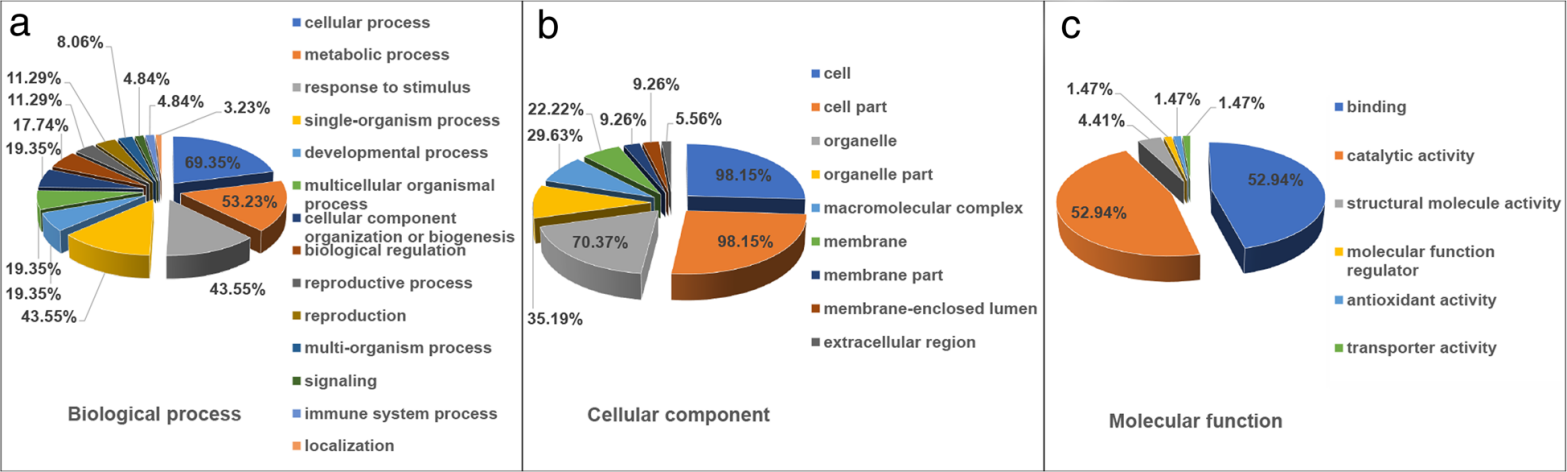
The distribution of DEPs involved in biological process, cellular component, and molecular function according to GO analysis

Sequence homology analysis showed that 83 DEPs (92.2% of all DEPs) were associated with at least one GO term and categorized into 29 functional groups, consisting of 14 groups in biological process, 9 groups in cellular component, and 6 groups in molecular function (Fig. 3; Additional file 5: Figure S3; Additional file 6: Table S3). The numbers of DEGs involved in biological process, cellular component, and molecular function were 62, 54, and 68, respectively. For biological process, the most DEPs were categorized into cellular process, accounting for 69.35%. Meanwhile, for cellular component, the DEPs were also mostly localized in cell and cell part groups, accounting for 98.15%, respectively. These results showed that the suppression of multi-ovary traits was mainly associated with the altered cell activity. In addition, the nuclear multi-ovary gene was suppressed by TZI cytoplasm, suggesting a retrograde signaling from organelles to the nucleus. Coincidentally, following cell and cell part, the most DEPs were categorized into organelle, of which 11 DEPs were located in chloroplast, and 3 DEPs were located in mitochondria. These results implied that chloroplast might play an important role in the cytoplasmic suppression of multi-ovary trait.

**Fig. 4:**
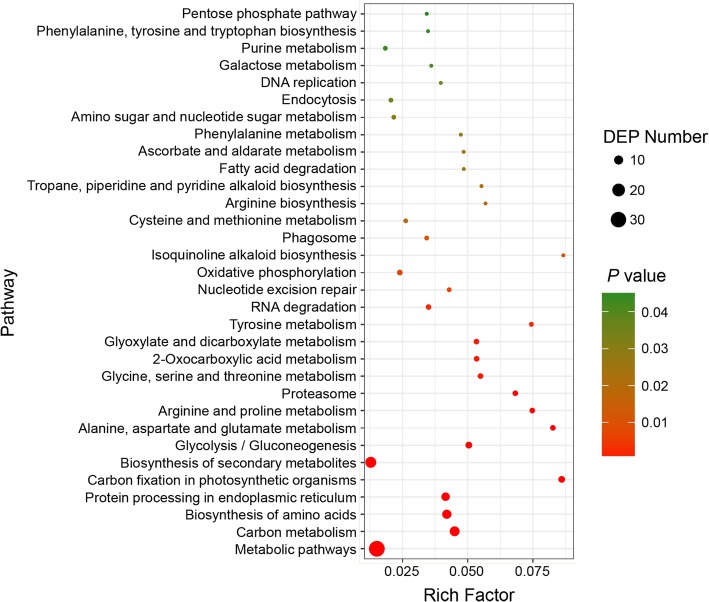
The KEGG enriched pathway of DEPs between TZI × DUOII and DUOII × TZI crosses. Rich factor is the ratio of the number of DEPs belonging to this pathway out of the number of proteins in the KEGG database belonging to this pathway. The size of circle represents the number of DEPs in this pathway, and the color represents the corrected *P* value, which is the significance level in enrichment analysis statistics. The enriched pathways were identified using a corrected *P* < 0.05. Details of DEPs in each pathway are listed in Additional file 7: Table S4

KEGG pathway analysis revealed that 63 terms were enriched for 57 DEPs (63% of all DEPs). Using a corrected *P* < 0.05, we identified 32 significantly enriched pathways (Fig. 4; Additional file 7: Table S4), consisting of 52 DEPs (91% of enriched DEPs), 12 upregulated and 40 downregulated. Detailed numbers of the upregulated and downregulated DEPs are shown in Additional file 8: Figure S4. For these significantly enriched pathways, there were not a relatively clear relationship between each other, and they were related to many different aspects and areas: such as carbon fixation in chloroplast, oxidative phosphorylation in mitochondria, protein processing in endoplasmic reticulum, pentose phosphate pathway and glycolysis in cytosol, DNA replication and repair in nuclear, and so on. These results suggested that a wide range of functional proteins were related to the process of heterogeneous cytoplasmic suppression of the multi-ovary trait and that the underlying mechanism of this phenomenon is a very complicated process.

### Bioinformatics-based PPI network analysis of identified DEPs

To explore the relationship between all the identified DEPs, a PPI network was created by blasting all the 90 DEPs against the *Arabidopsis* TAIR protein database. Identified proteins were grouped into functional classes according to their GO and KEGG analysis. The PPI network based on *Arabidopsis* homologs revealed five important functional groups principally involved in chloroplast metabolism, nuclear and cell division, plant respiration, protein metabolism, and flower development (Additional file 9: Figure S5). These five functional groups were not fully separated, but rather formed an interconnected network regulating the additional pistil development, which was mainly responsible for the heterogeneous cytoplasmic suppression of the multi-ovary trait. Abbreviations of the specific protein names in the PPI network are shown in Additional file 10: Table S5.

## Discussion

### Cytoplasm influences flower development in plants

The plant genome consists of both nuclear and cytoplasmic genomes, which cooperate to determine plant developmental process [32, 33]. Although the nuclear genome has a predominant role in determining the inheritance of most traits, the cytoplasmic genome and nuclear-cytoplasm interaction also play an essential role in plant flower development [34, 35]. In maize, the maternal cytoplasm can regulate the flowering time by interacting with nuclear genes [36]. In *Brassica*, the alloplasmic *Brassica oleracea* line with *Brassica rapa* cytoplasm exhibited shorter filament length, weakly developed anthers, smaller petal sizes and more than two stigmas [37]. In wheat, cytoplasmic male sterility is the most relevant cytoplasm-inherited trait and has been studied for many years [38, 39]. When the cytoplasm of Chinese Spring ditelosomic 7BS was replaced by *Aegilops crassa* cytoplasm, the alloplasmic line showed pistillody, and the pistils and transformed stamens were sterile due to abnormal ovule development, which was caused by the *Ae. crassa* cytoplasm affecting the expression of the wheat nuclear gene *WANT-1* [40]. In our study, DUOII × TZI F_1_ plants showed the multi-ovary trait, while TZI × DUOII F_1_ plants showed the normal mono-ovary trait. In theory, only the cytoplasm was different between these reciprocal crosses, and so the differences in the multi-ovary trait suggested that the special heterogeneous cytoplasm of TZI influenced the multi-ovary trait expression in wheat.

### Chloroplasts influence nuclear flower gene expression

Chloroplasts are the site of photosynthesis and multiple anabolic reactions essential for growth, development, and reproduction [41]. Signals from chloroplasts can modulate nuclear gene expression and regulate plant development, including flower development [42–44]. An *Arabidopsis* mutant, lacking the chloroplast-localized rhomboid protease, exhibited either a double stigma or a single stigma with distortions in shape and size [45]. In addition, chloroplasts in *Arabidopsis* function as essential sensors of high light, and transmit interorganellar retrograde signals to the nuclear-encoded flowering network, which regulates flowering and adaptive responses by triggering nuclear transcriptional changes at the chromatin level [43]. In the present study, we identified 15 DEPs located in the chloroplast (Additional file 11: Figure S6), and these DEPs were involved in chlorophyll synthesis (spot 60, glutamate-1-semialdehyde 2,1-aminomutase, chloroplastic), photosystem II stability (spot 32, photosystem II stability/assembly factor HCF136, chloroplastic), ribulose bisphosphate carboxylase (Rubisco) assembly (spot 17 and spot 36, Rubisco large subunit-binding protein subunit alpha; spot 38, chaperonin 60-like protein; spot 50 and spot 55, transketolase, chloroplastic; spot 86, Rubisco large chain), ATP synthesis (spot 37, ATP synthase CF1 beta subunit, chloroplastic), chloroplastic post-transcriptional gene expression (spot 1, 29 kDa ribonucleoprotein A, chloroplastic-like; spot 2, 31 kDa ribonucleoprotein, chloroplastic), and chloroplastic protein biosynthesis (spot 45, elongation factor Tu, chloroplastic; spot 56, carbamoyl-phosphate synthase large chain, chloroplastic; spot 68, ketol-acid reductoisomerase, chloroplastic; spot 75, chloroplast aspartate aminotransferase).

Chlorophyll synthesis, photosystem II stability, Rubisco assembly and ATP synthesis were different aspects of photosynthetic system and included nine DEPs, of which six were downregulated and three were upregulated in TZI × DUOII. These DEPs might disrupt the photosynthesis system and its related metabolism process in chloroplast. Spot 1 and spot 2 were identified as chloroplast ribonucleoprotein, which played a global role in post-transcriptional gene expression processes including editing and stability of specific chloroplast mRNA and rRNA [46–48]. These two chloroplast ribonucleoproteins were downregulated 2–3 fold in TZI × DUOII, which might cause this material to have low amounts of the key post-transcriptional regulators for processing RNA metabolism and plastid biogenesis. Among the four DEPs included in chloroplastic protein biosynthesis, three DEPs were involved in amino acid metabolism; and the altered amino acid metabolism could influence the biogenesis and activity of microRNAs, regulators in plant growth and development, via chloroplast-to-nucleus signaling [49].

The function of these DEPs showed that there was a big difference in the chloroplast metabolic and developmental processes between TZI × DUOII and DUOII × TZI. Although chloroplast development is largely under nuclear control, developmentally arrested or damaged chloroplast can regulate nuclear gene expression via retrograde signaling pathways [42]. So, in TZI × DUOII plants, these altered processes might transmit different signals to the nucleus and trigger nuclear transcriptional changes, probably resulting the suppression of additional pistil development.

### Nuclear and cell division are the basis of the differentiation of additional pistil primordium

Plant flowers arise from a specialized structure called the shoot apical meristem, which comprises a pool of stem cells that continuously divide and replenish [50]. The shoot apical meristem produces floral meristems, in which floral organ primordia are formed and developed into organs by coordinated cell division and differentiation [51]. As shown in Fig. 2, the additional pistil of the multi-ovary wheat was derived from a protuberance between the frontal stamen and lateral stamen. The primordium producing this additional pistil results from the abnormal division of a subcortical cell [3], confirming that disruption of nuclear and cell division can cause alterations in cell fate and pistil differentiation [52, 53]. In the present study, 18 DEPs, identified as 15 different proteins, were related to the nuclear and cell division process, with 17 DEPs downregulated and one upregulated in the TZI × DUOII plants (Additional file 11: Figure S6).

As shown in the PPI analysis (Additional file 9: Figure S5), VIP3 (Vernalization Independence 3, spot 73) was the protein connected to the flower development process. VIP3, a protein consisting almost exclusively of repeated Trp-Asp (WD) motifs, plays an essential role in the proper progression of the cell differentiation process. When the expression of VIP3 was disrupted, the plant showed specific defects in floral morphology due to the altered development of shoot apical meristems in *Arabidopsis* [54, 55]. Spot 81, the only upregulated DEP involved in nuclear and cell division in the TZI × DUOII plants, was identified as the COMPASS-like H3K4 histone methylase component WDR5A. In *Arabidopsis*, overexpression of WDR5A could inhibit floral transition by suppressing the differentiation of shoot apical meristem cells [56, 57]. Except for these two DEPs, the other 16 DEPs also played a vital role in nuclear and cell division, such as DNA polymerase alpha (spot 52) [58], replication protein A (spot 57) [59], and nucleosome assembly protein (spot 5) [60]. These DEPs suggested that heterogeneous cytoplasm could disrupt nuclear and cell division events, which might then inhibit the differentiation of additional pistil primordium.

### Plant respiration provides energy for additional pistil differentiation

All chemical reactions in living cells are energy dependent. In biological processes, energy drives the cellular metabolism and transport processes that are necessary for plant growth and developmental switching [61, 62]. Depending on the metabolic demands, production, transportation, utilization and conversion of energy within cells are typically dynamic and require the coordination of different organelles to achieve specific developmental switches [63]. Plant respiration usually involves the controlled oxidation of reduced carbohydrates via the sequential pathways of cytosolic glycolysis, the mitochondrial tricarboxylic acid cycle, and mitochondrial electron transport chain. Respiratory electron transfer through the mitochondrial electron transport chain releases a large amount of free energy of ATP, which serves as a universal energy source for various life activities in plants [64]. Energy metabolism plays a critical role in stem cell maintenance and differentiation, and abnormal energy metabolism can alter floral organ development in plants [65, 66]. In the present study, 16 DEPs were identified in the plant respiration process, with 14 downregulated and two upregulated (Additional file 11: Figure S6). So, energy metabolism was disrupted in the TZI × DUOII plants, which might not supply enough energy for the additional pistil differentiation and possibly resulted in the suppression of the multi-ovary trait.

### Protein metabolism is the basis of the metabolic processes involved in plant development

The normal development of a plant is dependent on the complex and precise interaction of different metabolic processes, in which, proteins are the main and direct executors of life functions and reflect the complex molecular and physiological processes [10, 11]. In this study, 19 DEPs were involved in protein metabolism (Additional file 11: Figure S6), and these DEPs were implicated in four aspects: amino acid synthesis, protein synthesis, protein assembling/folding, and protein degradation. All of these aspects ensure that the synthesized protein has a correct structure and amount, which is the basis of properly functioning metabolic process. If any one of these aspects is altered, the amount of correctly synthesized protein will be changed, which will then influence some metabolic processes in the plant [67, 68]. For example, the ubiquitin-proteasome pathway is a major route for selectively degrading cytoplasmic and nuclear proteins in eukaryotes. In plants, this pathway significantly contributes to development by affecting a wide range of processes, including cell-cycle progression, DNA repair, organelle biogenesis, signal transduction, programmed cell death, and communication between plastids and nucleus [69, 70]. In this study, six identified DEPs (spot 10, 26S protease regulatory subunit 6A-like protein A; spot 15, ubiquitin-like protein; spot 21, inversin-B; spot 23, ubiquitin domain-containing protein DSK2a-like isoform X2; spot 64, 26S protease regulatory subunit 6B-like protein; spot 79, proteasome subunit alpha type-6) were involved in the ubiquitin-proteasome pathway. These DEPs altered protein metabolism, which might have then disrupted some metabolic processes and suppressed multi-ovary trait expression in the TZI × DUOII plants.

### Proteins involved in flower development are crucial for multi-ovary trait expression

The normal development of floral organs relies on floral meristem homeostasis, proper organ specification and regional differentiation. These development processes are dependent on the intricate and precise regulation of gene products. In the present study, six DEPs were found to be associated with the flower development process through the heterogeneous cytoplasm suppression of the multi-ovary trait (Additional file 11: Figure S6). Of these, three DEPs (spot 25, spot 26, and spot 27) belonged to flowering locus K homology (KH) domains (FLK). FLK is an RNA-binding protein with three KH domains, and it plays an essential role in normal plant flowering. In *Arabidopsis*, when the expression of the *FLK* gene was inactivated, the floral transition was suppressed, and mutants exhibited severe late-flowering phenotypes [71, 72]. Similar to FLK, spot 51 was identified as another RNA-binding KH domain-containing protein PEPPER (PEP), which acted on vegetative growth and pistil development. In *Arabidopsis*, the *PEP* gene was initially identified in a mutant with aberrant phyllotaxy and small fruits with supernumerary carpels. PEP and FLK interacted to regulate the flowering time by influencing the expression of *FLOWERING LOCUS C*, a central repressor of flowering time [73, 74]. Meanwhile, PEP and FLK also played a vital role in the specification of flower organ identity by post-transcriptionally regulating the MADS-box floral homeotic gene *AGAMOUS* (*AG*) in *Arabidopsis* [75]. *AG*, a C-function gene of the ABC model, specifies female carpels and defines the pistil or gynoecium situated in the innermost whorl. In wheat, the product of an *AG* homolog, *WAG*, is involved in pistil development and is associated with pistillody caused by a nuclear-cytoplasm interaction in alloplasmic wheat [76].

Unlike with FLK and PEP, the other two DEPs (spot 53, spot 67), closely connected to FLK and PEP in the PPI analysis, were not directly associated with pistil development. Spot 53 was identified as the far-upstream element-binding protein 2 (FBP2), which was a member of the single-stranded DNA-binding protein family. FBP2 was correlated with cell cycle progression by accelerating the G1/S transition, and the knockdown of FBP2 could weaken stem cell proliferation. In addition, phosphorylated FBP2 could shuttle from the cytoplasm into the nucleus, which is the basis of cytoplasm-nuclear interactions [77]. Spot 67 was identified as polyadenylate-binding protein RBP45, which was a member of the heterogeneous nuclear ribonucleoprotein (hnRNP)-protein binding poly(A) tail of mRNA. RBP45 was localized in the nucleus and probably involved in some steps of pre-mRNA maturation [78].

In the present study, all six of these DEPs were downregulated in the TZI × DUOII plants compared to the DUOII × TZI plants. According to their functions, downregulated RBP45 and FBP2 interacted to weaken stem cell proliferation, which might be responsible for the lack of additional pistil protuberance. Meanwhile, in regards to the role of PEP and PLK in the specification of pistil organ identity, their downregulation might reduce the expression of AG, which possibly then influenced the production of additional pistils in alloplasmic wheat. Taken together, these DEPs might play an essential role in the underlying mechanism that controls the heterogeneous cytoplasm-induced suppression of the nuclear multi-ovary gene in wheat.

## Conclusions

DUOII is an excellent variety of wheat for studying the mechanisms of the multi-ovary trait and floral development. The multi-ovary trait of DUOII is controlled by a dominant gene, which is suppressed by the heterogeneous cytoplasm of TZI. Although the nuclear genetic control of DUOII is clear, little is known about the underlying mechanism of heterogeneous cytoplasmic suppression of the multi-ovary trait in common wheat. Here, we applied a proteomics approach to attempt to identify key proteins involved in the cytoplasmic suppression of the multi-ovary trait in wheat. The comparative protein profiles were characterized to identify proteins differentially regulated in the plants with the TZI cytoplasm to give insight to proteins that may play a role in suppressing the multi-ovary trait. Our results showed that the suppression of the multi-ovary trait was associated with DEPs during the critical stage of additional pistil primordium development (2–6 mm long young spikes). These proteins were mainly involved in chloroplast metabolism, nuclear and cell division, plant respiration, protein metabolism, and flower development. In the TZI × DUOII plants, we proposed that the different chloroplast metabolism and development might transmit different signals to the nucleus and trigger nuclear transcriptional changes, probably resulting in the altered nuclear and cell division events, which subsequently suppressed the differentiation of additional pistil primordium. In this process, the altered plant respiration and protein metabolism could not supply enough energy and correct proteins for the additional pistil differentiation. In addition, we identified two key proteins, PEP and FLK, which might play an essential role in the specification of pistil organ identity. Overall, this paper provided a universal proteomic profiling involved in the cytoplasmic suppression of wheat floral meristems, and our results have laid a solid foundation for further mechanistic studies on the underlying mechanisms that control the heterogeneous cytoplasm-induced suppression of the nuclear multi-ovary gene in wheat.

## Additional files

Additional file 1: **Figure S1.** 2-DE analysis of proteins extracted from TZI × DUOII and DUOII × TZI young spikes. About 900 μg of protein sample was loaded on each IPG strip (pH 4–7), and the gels were visualized with Coomassie brilliant blue G250 solution. The experiment was repeated three times, and the three gels under each heading represent three replications. The identified differentially expressed proteins are labeled on the gels. (DOCX 561 kb)

Additional file 2: **Figure S2.** Volcano plots showing DEPs in TZI × DUOII cross relative to DUOII × TZI. (DOCX 122 kb)

Additional file 3: **Table S1.** Identification of differentially expressed proteins between DUOII × TZI and TZI × DUOII. (XLSX 24 kb)

Additional file 4: **Table S2.** Corresponding homologues of the predicted proteins and unnamed protein. (XLSX 16 kb)

Additional file 5: **Figure S3.** GO classification of DEPs in TZI × DUOII relative to DUOII × TZI. Details of GO classification are listed in Additional file 6: Table S3. (DOCX 470 kb)

Additional file 6: **Table S3.** GO classification of differentially expressed proteins. (XLSX 11 kb)

Additional file 7: **Table S4.** Significantly enriched KEGG pathways of differentially expressed proteins. (XLSX 12 kb)

Additional file 8: **Figure S4.** Histogram of differentially expressed proteins (DEPs) involved in the significantly enriched KEGG pathway. The Y axis represents the KEGG pathway term, and the X axis represents the number of DEPs. The red bars and greens bars indicate the number of upregulated and downregulated DEPs, respectively. (DOCX 457 kb)

Additional file 9: **Figure S5.** Protein interaction network analysis using STRING 10.0. DEPs were mapped to *Arabidopsis thaliana* homologs by searching the STRING 10.0 databases with a confidence cutoff of 0.4. The proteins are the supposed orthologs of the DEPs in TZI × DUOII. Colored lines between the proteins indicate the type of interaction evidence. Details of all the protein nodes are listed in Additional file 10: Table S5. (DOCX 1194 kb)

Additional file 10: **Table S5**. Protein interaction network analysis by searching the STRING 10.0 according to TAIR homologous proteins. (XLSX 15 kb)

Additional file 11: **Figure S6.** Heat map of DEPs of categories classified in the PPI analysis. The heat map analysis was conducted with the transformed log1.5 of fold change ratios. The numbers were spot numbers correspond with 2-D gel as shown in Additional file 1: Figure S1. A, Chloroplast metabolism; B, Nuclear and cell division; C, Plant respiration; D, Protein metabolism; E, Flower development; F, Other. (DOCX 284 kb)

## Abbreviations

2-DE: Two-dimensional electrophoresis
2-ME: 2-mercaptoethanol
ABC: Ammonium bicarbonate
ACN: Acetonitrile
AG: AGAMOUS
CBB: Coomassie brilliant blue
CHAPS: 3-[(3-cholamidopropyl) dimethylammonio] propanesulfonate
DEPs: Differentially expressed proteins
DTT: DL-dithiothreitol
FA: Formic acid
FBP2: Far-upstream element-binding protein 2
FLK: Flowering locus KH domain
GO: Gene Ontology
HCD: High-energy collision-induced dissociation
hnRNP: heterogeneous nuclear ribonucleoprotein
HPLC: High performance liquid chromatography
IEF: Isoelectric focusing
KEGG: Kyoto Encyclopedia of Genes and Genomes
KH: K homology
LC-HESI-MS/MS: Liquid chromatography with heated electrospray ionization tandem mass spectrometry
LSD: Least-significant difference
Mr.: Molecular weight
MS: Mass spectrometry
PEP: PEPPER
pI: Isoelectric point
PMSF: Phenylmethanesulfonyl fluoride
PPI: Protein-protein interaction
Rubisco: Ribulose bisphosphate carboxylase
SDS: sodium dodecyl sulfate
SDS-PAGE: Sodium dodecyl sulfate-polyacrylamide gel electrophoresis
TCA: Trichloroacetic acid
TZI: TeZhiI
VIP3: Vernalization Independence 3
