# Supplementary material for: Comparative proteomic analysis of multi-ovary wheat under heterogeneous cytoplasm suppression
Source: BMC Plant Biol. 2019 May 2;19:175. doi: 10.1186/s12870-019-1778-y (PMC6498644; doi:10.1186/s12870-019-1778-y)
Supplement: Supplementary file 1 — Figure S1. 2-DE analysis of proteins extracted from TZI × DUOII and DUOII × TZI young spikes. About 900 μg of protein sample was loaded on each IPG strip (pH 4–7), and the gels were visualized with Coomassie brilliant blue G250 solution. The experiment was repeated three times, and the three gels under each heading represent three replications. The identified differentially expressed proteins are labeled on the gels. (DOCX 561 kb) [file 12870_2019_1778_MOESM1_ESM.docx]

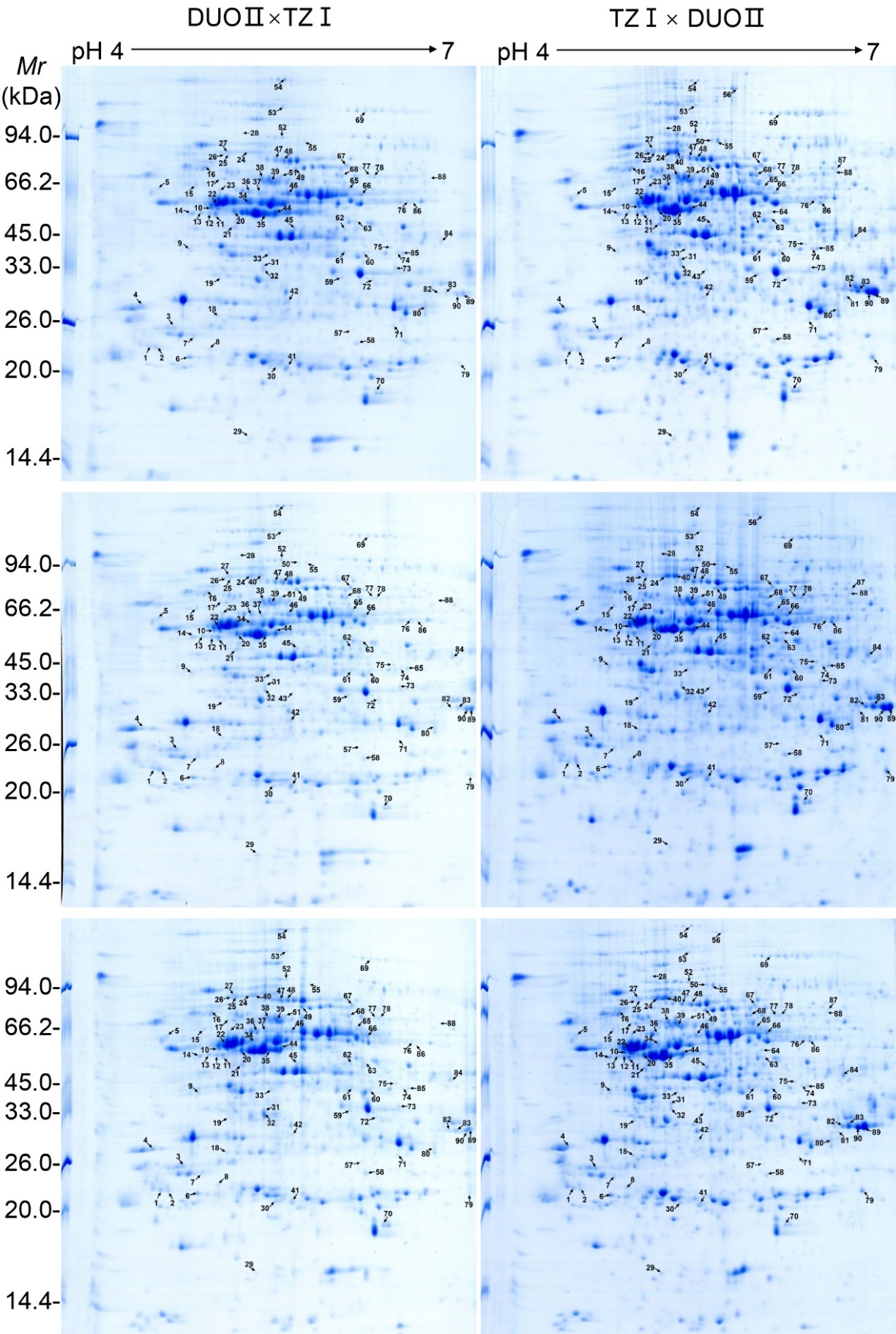


**Figure S1** 2-DE analysis of proteins extracted from TZI × DUOII and DUOII × TZI young spikes. About 900 μg of protein sample was loaded on each IPG strip (pH 4–7), and the gels were visualized with Coomassie brilliant blue G250 solution. The experiment was repeated three times, and the three gels under each heading represent three replications. The identified differentially expressed proteins are labeled on the gels.
