# Supplementary figures and images for: Comparative proteomic analysis of multi-ovary wheat under heterogeneous cytoplasm suppression
Source: BMC Plant Biol. 2019 May 2;19:175. doi: 10.1186/s12870-019-1778-y (PMC6498644; doi:10.1186/s12870-019-1778-y)

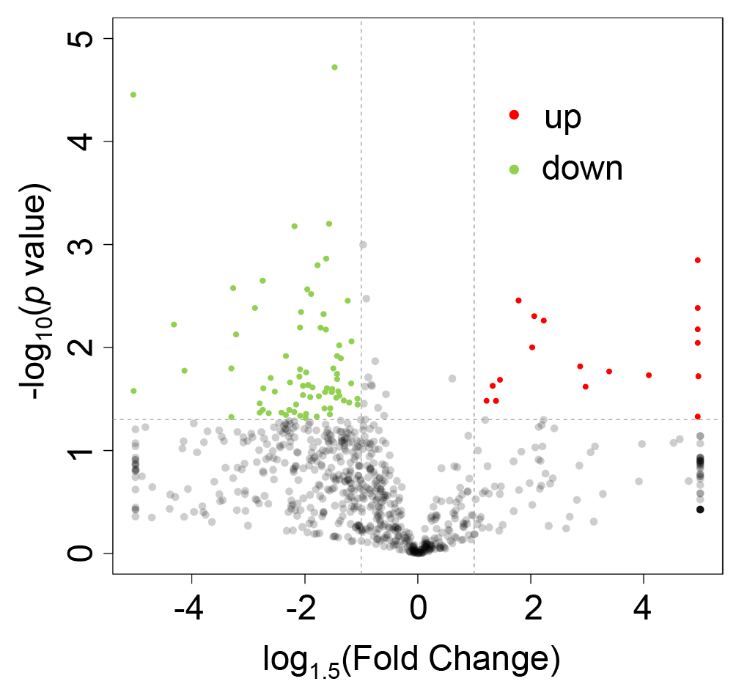


**Figure S2** Volcano plots showing DEPs in TZI × DUOII cross relative to DUOII × TZI.

Supplement: Supplementary file 2 — Figure S2. Volcano plots showing DEPs in TZI × DUOII cross relative to DUOII × TZI. (DOCX 122 kb) [file 12870_2019_1778_MOESM2_ESM.docx]
