# Supplementary material for: Comparative proteomic analysis of multi-ovary wheat under heterogeneous cytoplasm suppression
Source: BMC Plant Biol. 2019 May 2;19:175. doi: 10.1186/s12870-019-1778-y (PMC6498644; doi:10.1186/s12870-019-1778-y)
Supplement: Supplementary file 5 — Figure S3. GO classification of DEPs in TZI × DUOII relative to DUOII × TZI. Details of GO classification are listed in Additional file 6: Table S3. (DOCX 470 kb) [file 12870_2019_1778_MOESM5_ESM.docx]

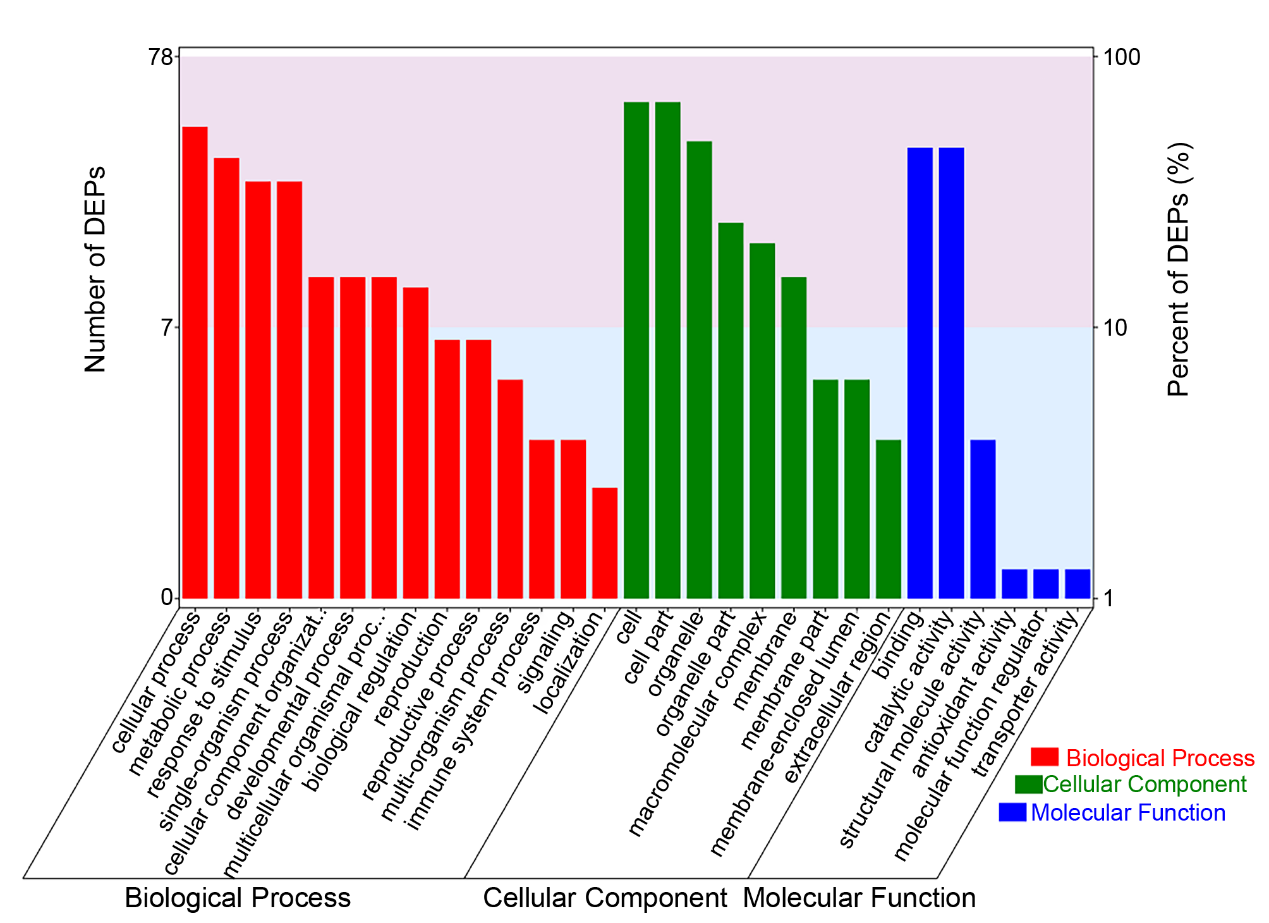


**Figure S3** GO classification of DEPs in TZI × DUOII relative to DUOII × TZI. Details of GO classification are listed in Additional file 6: Table S3.
