# Supplementary material for: Comparative proteomic analysis of multi-ovary wheat under heterogeneous cytoplasm suppression
Source: BMC Plant Biol. 2019 May 2;19:175. doi: 10.1186/s12870-019-1778-y (PMC6498644; doi:10.1186/s12870-019-1778-y)
Supplement: Supplementary file 8 — Figure S4. Histogram of differentially expressed proteins (DEPs) involved in the significantly enriched KEGG pathway. The Y axis represents the KEGG pathway term, and the X axis represents the number of DEPs. The red bars and greens bars indicate the number of upregulated and downregulated DEPs, respectively. (DOCX 457 kb) [file 12870_2019_1778_MOESM8_ESM.docx]

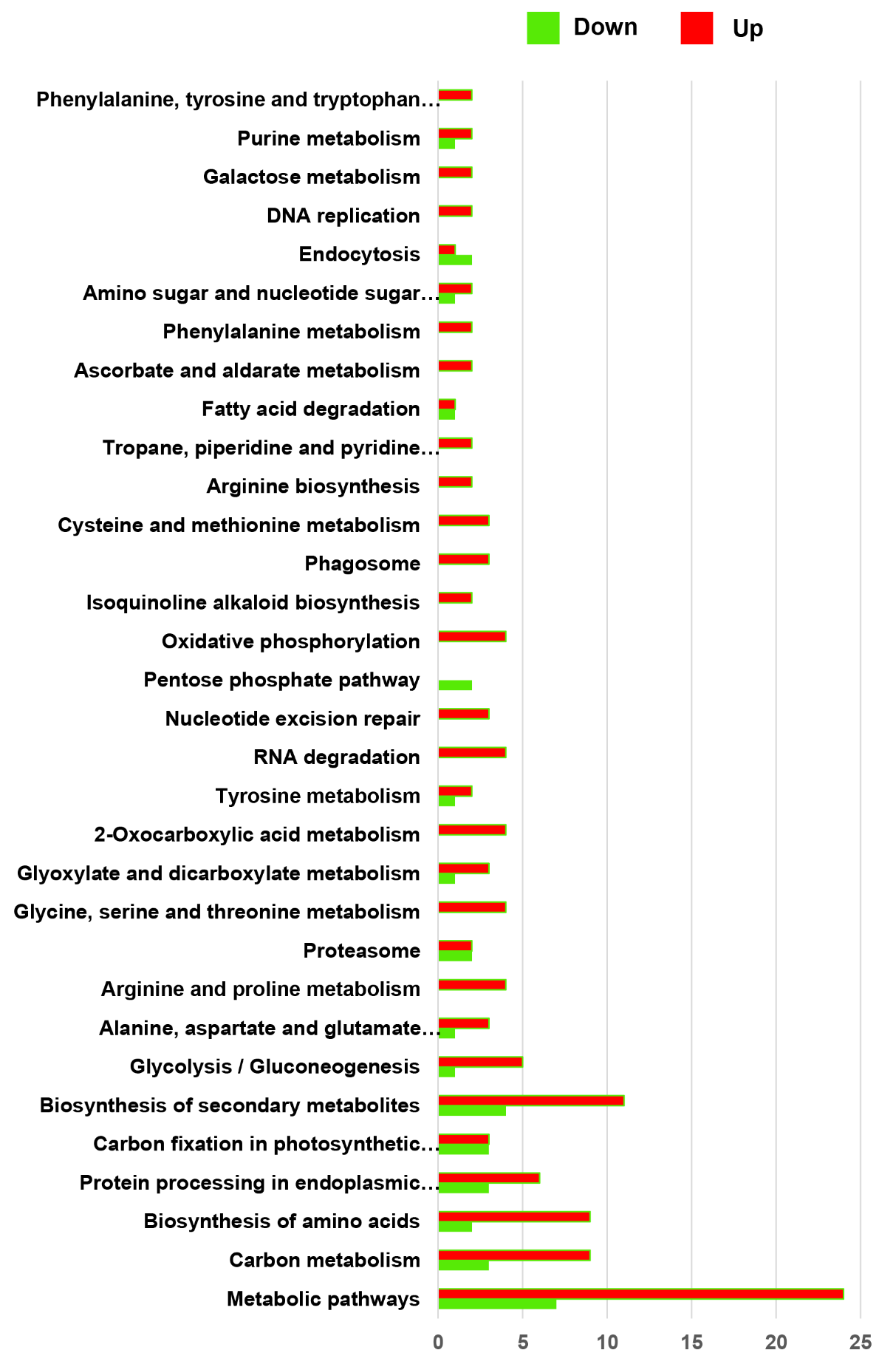


**Figure S4** Histogram of differentially expressed proteins (DEPs) involved in the significantly enriched KEGG pathway. The Y axis represents the KEGG pathway term, and the X axis represents the number of DEPs. The red bars and greens bars indicate the number of upregulated and downregulated DEPs, respectively.
