# Supplementary material for: Comparative proteomic analysis of multi-ovary wheat under heterogeneous cytoplasm suppression
Source: BMC Plant Biol. 2019 May 2;19:175. doi: 10.1186/s12870-019-1778-y (PMC6498644; doi:10.1186/s12870-019-1778-y)
Supplement: Supplementary file 9 — Figure S5. Protein interaction network analysis using STRING 10.0. DEPs were mapped to Arabidopsis thaliana homologs by searching the STRING 10.0 databases with a confidence cutoff of 0.4. The proteins are the supposed orthologs of the DEPs in TZI × DUOII. Colored lines between the proteins indicate the type of interaction evidence. Details of all the protein nodes are listed in Additional file 10: Table S5. (DOCX 1194 kb) [file 12870_2019_1778_MOESM9_ESM.docx]

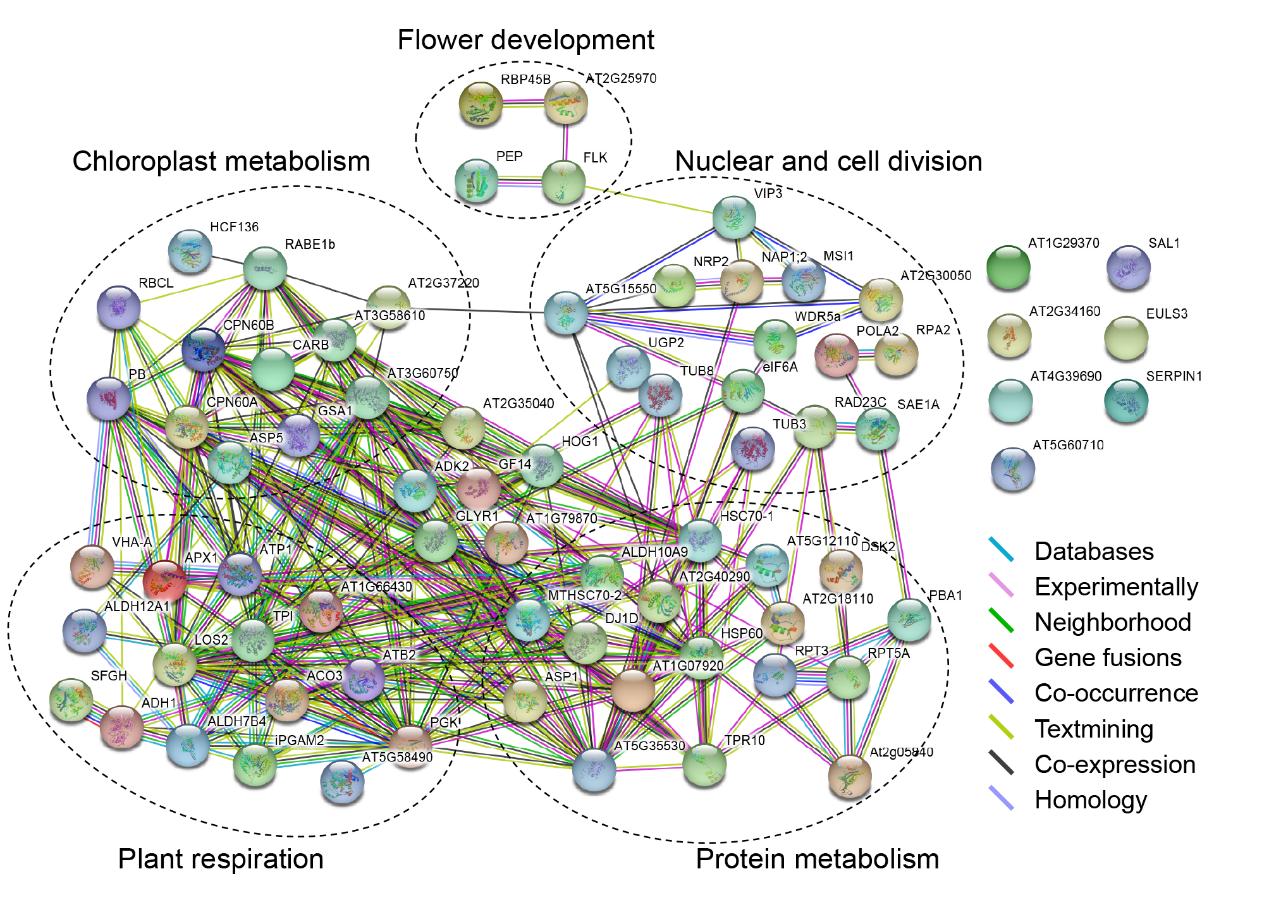


**Figure S5** Protein interaction network analysis using STRING 10.0. DEPs were mapped to *Arabidopsis thaliana* homologs by searching the STRING 10.0 databases with a confidence cutoff of 0.4. The proteins are the supposed orthologs of the DEPs in TZI × DUOII. Colored lines between the proteins indicate the type of interaction evidence. Details of all the protein nodes are listed in Additional file 10: Table S5.
