# Supplementary material for: Comparative proteomic analysis of multi-ovary wheat under heterogeneous cytoplasm suppression
Source: BMC Plant Biol. 2019 May 2;19:175. doi: 10.1186/s12870-019-1778-y (PMC6498644; doi:10.1186/s12870-019-1778-y)
Supplement: Supplementary file 11 — Figure S6. Heat map of DEPs of categories classified in the PPI analysis. The heat map analysis was conducted with the transformed log1.5 of fold change ratios. The numbers were spot numbers correspond with 2-D gel as shown in Additional file 1: Figure S1. A, Chloroplast metabolism; B, Nuclear and cell division; C, Plant respiration; D, Protein metabolism; E, Flower development; F, Other. (DOCX 284 kb) [file 12870_2019_1778_MOESM11_ESM.docx]

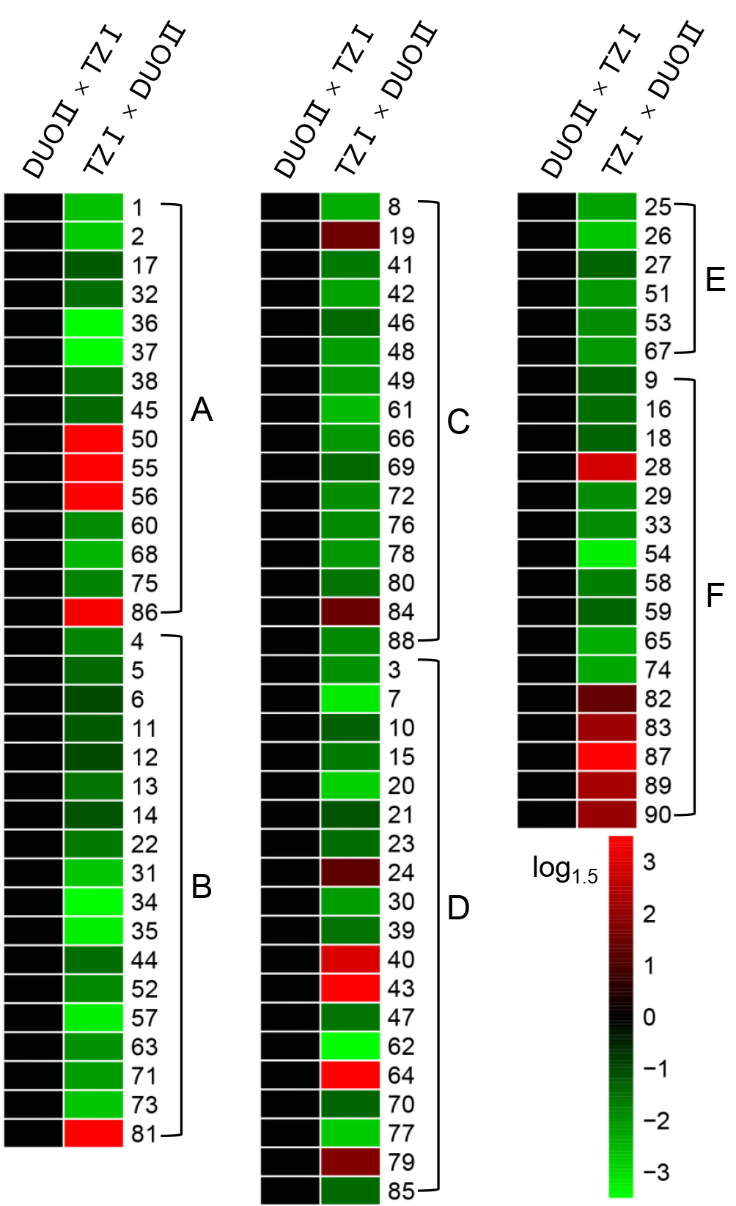


**Figure S6** Heat map of DEPs of categories classified in the PPI analysis. The heat map analysis was conducted with the transformed log1.5 of fold change ratios. The numbers were spot numbers correspond with 2-D gel as shown in Additional file 1: Figure S1. A, Chloroplast metabolism; B, Nuclear and cell division; C, Plant respiration; D, Protein metabolism; E, Flower development; F, Other.
